# Evaluation of a breastmilk hand expression toolkit: the M.I.L.K survey study

**DOI:** 10.1186/s13006-021-00448-3

**Published:** 2022-01-15

**Authors:** Kameela Miriam Alibhai, Malia S. Q. Murphy, Sandra Dunn, Erin Keely, Paloma O’Meara, Josdalyne Anderson, Darine El-Chaâr

**Affiliations:** 1 Faculty of Medicine, University of Ottawa, Ottawa, Canada; 2 OMNI Research Group, Clinical Epidemiology Program, Ottawa Hospital Research Institute, Box 241, 501 Smyth Rd, Ottawa, ON K1H 8L6 Canada; 3 BORN Ontario, Children’s Hospital of Eastern Ontario, Ottawa, Canada; 4 CHEO Research Institute, Ottawa, Canada; 5 School of Nursing, University of Ottawa, Ottawa, Canada; 6 Division of Endocrinology and Metabolism, The Ottawa Hospital, Ottawa, Canada; 7 Department of Medicine, University of Ottawa, Ottawa, Canada; 8 Division of General Internal Medicine, The Ottawa Hospital, Ottawa, Canada; 9 Division of Maternal and Newborn Care, The Ottawa Hospital, Ottawa, Canada; 10 Department of Family Medicine, University of Ottawa, Ottawa, Canada; 11 Division of Maternal-Fetal Medicine, The Ottawa Hospital, Ottawa, Canada; 12 Department of Obstetrics and Gynecology, University of Ottawa, Ottawa, Canada; 13 School of Epidemiology and Public Health, University of Ottawa, Ottawa, Canada

**Keywords:** Breastfeeding, Breastmilk, Colostrum, Hand expression, Patient education

## Abstract

**Background:**

Breastmilk hand expression (BMHE) is recommended to promote lactation, relieve breast engorgement, and collect milk for future infant feedings. Resources to teach this skill are limited and infrequently developed in partnership with the obstetrical population. In collaboration with maternity care experts and individuals with recent breastfeeding experience, we designed a one-page toolkit that describes the process of BMHE and includes step-by-step instructions and images to illustrate the technique. This study aimed to evaluate the readability, clarity of content, layout, and informational value of this BMHE toolkit.

**Methods:**

Individuals who intended to breastfeed, were currently breastfeeding, or had recently breastfed were electronically surveyed and completed a two-part survey that consisted of radio, multi-select, Likert scale, and open-ended questions. Part one captured sociodemographic factors, obstetrical history, and breastfeeding practices. Part two collected feedback on the BMHE toolkit. Participants were recruited electronically through social media and posters were circulated in antenatal and postnatal care settings in Ottawa, Canada between November 2020 and February 2021.

**Results:**

Of the 123 participants, 117 (95.1%) had heard of hand expression prior to reviewing the toolkit and 99 (80.5%) had hand expressed before. Among the 48 participants who were no longer exclusively breastfeeding at the time of the survey, 22 (45.8%) had exclusively breastfed their infant for at least six months and 7 (14.6%) had discontinued exclusive breastfeeding within the first month. When asked about the BMHE toolkit, 118 (95.9%) participants said it was informative, 115 (93.5%) said it was easy to understand, and 114 (92.7%) said it was well laid-out. When asked about information seeking behaviours, participants indicated a preference for online resources (58.5%) and video resources (22.0%).

**Conclusions:**

The BMHE toolkit was well received by participants and the feedback was favourable overall. The survey feedback will be used to create a revised version of the toolkit that has been validated by the obstetrical patient population. Future research should focus on identifying implementation strategies to optimize the use of the toolkit and increase its effectiveness as an educational resource to teach participants correctly BMHE.

**Supplementary Information:**

The online version contains supplementary material available at 10.1186/s13006-021-00448-3.

## Background

The World Health Organization currently recommends that pregnant individuals exclusively breastfeed their infant for the first six months of life and subsequently introduce complementary solid foods in combination with breastmilk until children are at least two years old [1]. Exclusive breastfeeding involves feeding an infant human breastmilk and only providing medications, vitamins and oral rehydration when necessary [2]. The health benefits of breastfeeding, for both mother and infant, have been studied extensively [3–10]. However, initiation and maintenance of breastfeeding after delivery can be challenging for many women [11], and is influenced by cultural, psychological, and physiological factors [12]. Approximately 25% of individuals cease breastfeeding before their infant is one-month old and approximately only 8% of Canadian individuals continue to exclusively breastfeed at six months of age [13, 14]. Gionet et al. (2013) found that perceived breastmilk insufficiency and the belief that the infant is not receiving enough milk were the two most cited reasons for early weaning within the first month after delivery [15].

Breastmilk hand expression (BMHE) is a technique that involves massaging the breast to stimulate the mammary glands to release breastmilk. This technique can be used to promote lactation, soften to assist latching, relieve painful breast engorgement, and collect milk for future infant feedings [16, 17]. Hand expression during the antenatal period may also be recommended to improve mothers’ breastfeeding confidence before delivery [18, 19] and collect colostrum for use for infant feedings shortly after delivery. Antenatal colostrum collection has been recommended to individuals with diabetes who have low risk pregnancies to support infant feeds in the early hours and days post-delivery, prevent neonatal hypoglycemia and reduce the need for infant formula use [20]. The current literature suggests that individuals who hand express breastmilk antenatally may encounter fewer challenges when initiating breastfeeding and are less likely to discontinue breastfeeding before six months [16, 21].

Patient education materials should be evaluated by the target audience, made accessible to all patients regardless of education level and language. Although BMHE may be a viable method to improve breastfeeding outcomes [18, 22], there are limited resources to teach individuals this technique [23]. Additionally, available resources are often written with language exceeding seventh grade reading levels, contain complex medical terminology and infrequently developed in partnership with the obstetrical population [24–26]. The objective of this study was to collect individuals’ feedback regarding the readability, clarity of content, layout, and informational value of a newly developed BMHE toolkit developed in consultation with clinicians who work with the obstetrical population and individuals with recent breastfeeding experience. We also sought to determine participants’ information seeking behaviour patterns and preferences for breastfeeding and pregnancy resources.

## Methods

### Study design

This was an online survey study conducted to evaluate a novel, educational BMHE toolkit among pregnant individuals who intended to breastfeed and individuals who were currently breastfeeding or had stopped breastfeeding within the past year. Individuals who were 18 years or older, and able to understand English or French were eligible to participate in this study. The eligibility criteria were designed to capture input from individuals across multiple clinical care settings, patient sub-populations, and times in pregnancy. This is important as individuals’ perspectives may change depending on their proximity to the breastfeeding period. The survey and BMHE toolkit were made available to participants in both English and French using LimeSurvey, an online website platform [27]. Participation in the survey was entirely voluntary and no incentives were provided.

To facilitate participant recruitment, two distribution strategies were employed. First, electronic recruitment posters were shared on social media (e.g., Twitter, Facebook, and Instagram) through the personal and professional accounts of the study team. Recruitment materials were also posted in support groups that pregnant or recently pregnant individuals frequent, such as the Dr. MILK Facebook group composed of over 30,000 physicians who are interested in lactation knowledge. Second, recruitment posters were posted in antenatal and postnatal care settings of the General and Civic campuses of The Ottawa Hospital. Posters were also affixed on the clinic walls of The Monarch Centre sites, a multidisciplinary maternal and newborn health clinic in Ottawa, Canada. To minimize contact and reduce the spread of germs associated with paper-based surveys during the COVID-19 **pandemi**c, interested participants were able to scan a Quick Response (QR) code on the recruitment poster using their personal electronic device to review the hand expression toolkit and then complete the survey. Informed consent was implied by completion of the survey by the participant.

### Participant survey

The open, anonymous survey consisted of two-parts which each included dichotomous (yes/no), multiple choice, Likert scale and open-ended questions (see Additional file 1). In part one, participants were asked about their sociodemographic information, obstetrical history and current or recent experience with breastfeeding. In part two, participants were provided with a link to view the novel hand expression toolkit and were asked to answer questions regarding the toolkit’s overall readability, clarity of content, layout, and usefulness. Participants were also asked to provide section-specific feedback on the layout and complexity of the information enclosed in the toolkit. At the end of each section, participants were provided with a free text box to leave written feedback.

### BMHE toolkit

**Fig. 1:**
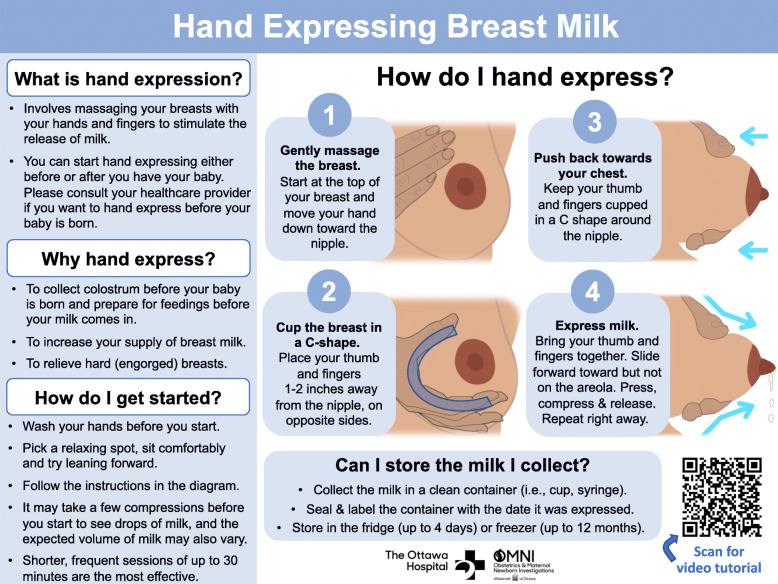
Breastmilk hand expression toolkit. The version depicted represents the final version after survey participant feedback was incorporated

The BMHE toolkit, depicted in Fig. 1, is a one-page educational resource that provides key information about hand expression. It was developed in consultation with an expert working group composed of a family physician who is also an internationally board-certified lactation consultant, a maternal-fetal-medicine specialist, a general internist, an endocrinologist and four individuals with recent breastfeeding experience. The toolkit is composed of four different sections. Section 1 (*What is hand expression?)* briefly explains what hand expression is, Section 2 (*Why hand express?*) provides reasons one may choose to hand express, Section 3 (*How do I get started?)* details how to prepare oneself to carry out BMHE, and Section 4 (*How do I hand express?)* includes step-by step instructions and detailed images illustrating the technique. The bottom right-hand corner of the toolkit contains a hyperlink to a seven-minute narrated video demonstrating BMHE to help individuals better understand this technique. The video, developed by Global Health Media, is freely accessible and available in several different languages, including English and French [28, 29]. The BMHE toolkit scored an 81.5/100 using the Flesch Reading Ease Formula, which suggests it is “Easy” to read, and suitable reading material for individuals with a sixth grade reading level or higher [30].

### Statistical analysis

We sought to obtain 150 survey responses excluding incomplete responses which were not eligible for analysis. Descriptive statistics were used to describe participant sociodemographic and obstetric characteristics. Categorical variables were summarized using frequencies and percentages and continuous variables were described using means and standard deviations (SD). All written feedback was qualitatively analyzed using an inductive coding approach [31]. First, one study author (KA) read all survey feedback and studied each response to identify common themes and categories. The findings were then discussed with two members of the study team (MM, DEC) to consider alternate meanings and finalize the coding frame. If a new theme was identified, each response was reread, and the coding frame was modified accordingly. Each participant’s written feedback was inductively coded using five emerging themes.

## Results

A total of 299 individuals clicked on the link to the survey. Of that, 210 (70.2%) individuals started filling out the survey, however, only 123 (41.1%) submitted the survey in its entirety. Of the 123 participants, the majority were currently breastfeeding (71.5%), Caucasian (85.4%), English-speaking (97.6%), multiparous (87.0%), had completed a graduate or professional degree (57.7%) and lived in the Ottawa/Gatineau region (53.7%). The average age of participants was 33 years. Additional participant characteristics are displayed in Table 1.

**Table 1:** Participant characteristics

Characteristics	***n*** (%)
Total number of individuals	123 (100)
Breastfeeding status	
Currently pregnant with intention of breastfeeding	25 (20.3)
Currently breastfeeding	88 (71.5)
Breastfed within the past year	10 (8.1)
Participant’s place of residence	
Ottawa/Gatineau region	66 (53.7)
Outside of Ottawa/Gatineau, but in Ontario	37 (30.1)
Outside of Ontario, but in Canada	9 (7.3)
Outside of Canada	10 (8.1)
Missing	1 (0.8)
Participant’s age (mean ± SD), years	33.0 ± 3.7
Participant’s ethnic or cultural group^a^	
Caucasian	105 (85.4)
Chinese	7 (5.7)
Arab	5 (4.1)
Black	3 (2.4)
Indigenous	2 (1.6)
Korean	2 (1.6)
Filipino	1 (0.8)
Southeast Asian	2 (1.6)
Latin American	1 (0.8)
Most commonly spoken or read languages by participants^a^	
English	120 (97.6)
French	24 (19.5)
Other	17 (13.8)
Participants highest level of education completed	
High school	4 (3.3)
College diploma/undergraduate degree	46 (37.4)
Graduate/Professional school	71 (57.7)
Prefer not to answer	2 (1.6)
Number of living children	
0	15 (12.2)
≥ 1	107 (87.0)
Missing	1 (0.8)

^a^This was a multi-select field, individuals could select multiple options. Percentages do not add to 100%. All data are presented as *n* (%) unless otherwise indicated

### Participants’ breastfeeding practices

**Fig. 2:**
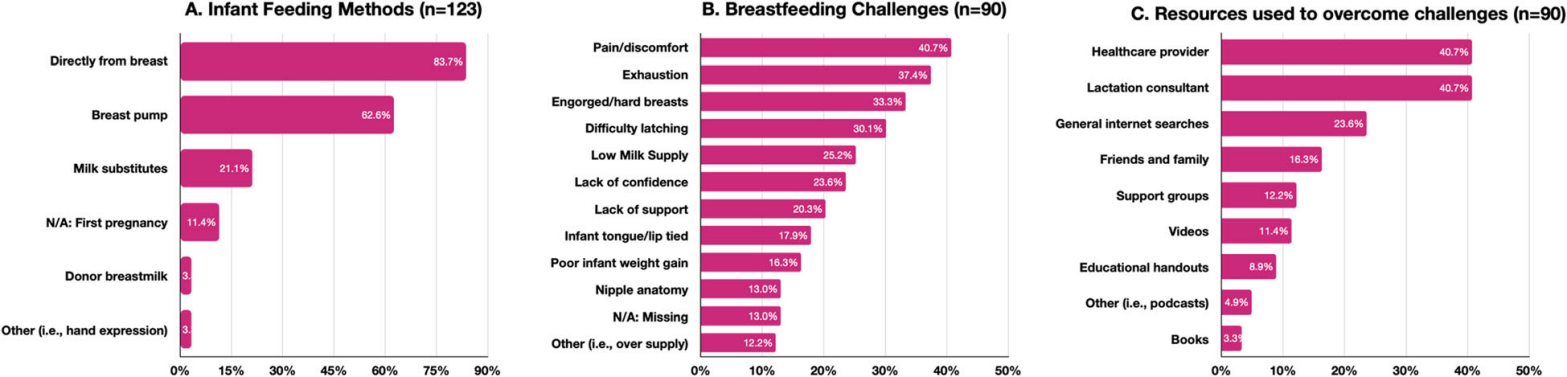
**A** All participants (*n* = 123) indicated the infant feeding method(s) they utilized. **B** Participants who had experience breastfeeding (*n* = 98) indicated what challenges they faced. **C** Participants who indicated they had faced breastfeeding challenges (*n* = 90) identified the types of resources they consulted

The most common infant feeding method was milk provided to the infant directly from the breast, with a total of 103 (83.7%) participants. A total of 98 participants had experience breastfeeding and of them 50 (51.0%) were exclusively breastfeeding at the time of the survey. Among the remaining 48 participants, 22 (45.8%) had exclusively breastfed their infant for at least six months and 7 (14.6%) had discontinued exclusive breastfeeding within the first month. The second most common infant feeding method was milk collected through a breast pump, which a total of 77 (62.6%) participants used. All 77 participants reported using the breast pump at least once a day. When asked about reasons for use of the pump, 61 (49.6%) of those participants reported using it in order to have a supply of milk for the infant while they were away and 26 (21.1%) reported using it because of low milk supply. A total of 90 (73.2%) participants reported facing challenges while breastfeeding; pain and discomfort, exhaustion, and engorged breasts were the three most cited challenges. Participants who reported facing challenges (*n* = 90) consulted healthcare practitioners and lactational consultants equally as often to try to overcome the challenges. These findings are depicted in Fig. 2.

### Participants’ perceptions of the BMHE toolkit

**Fig. 3:**
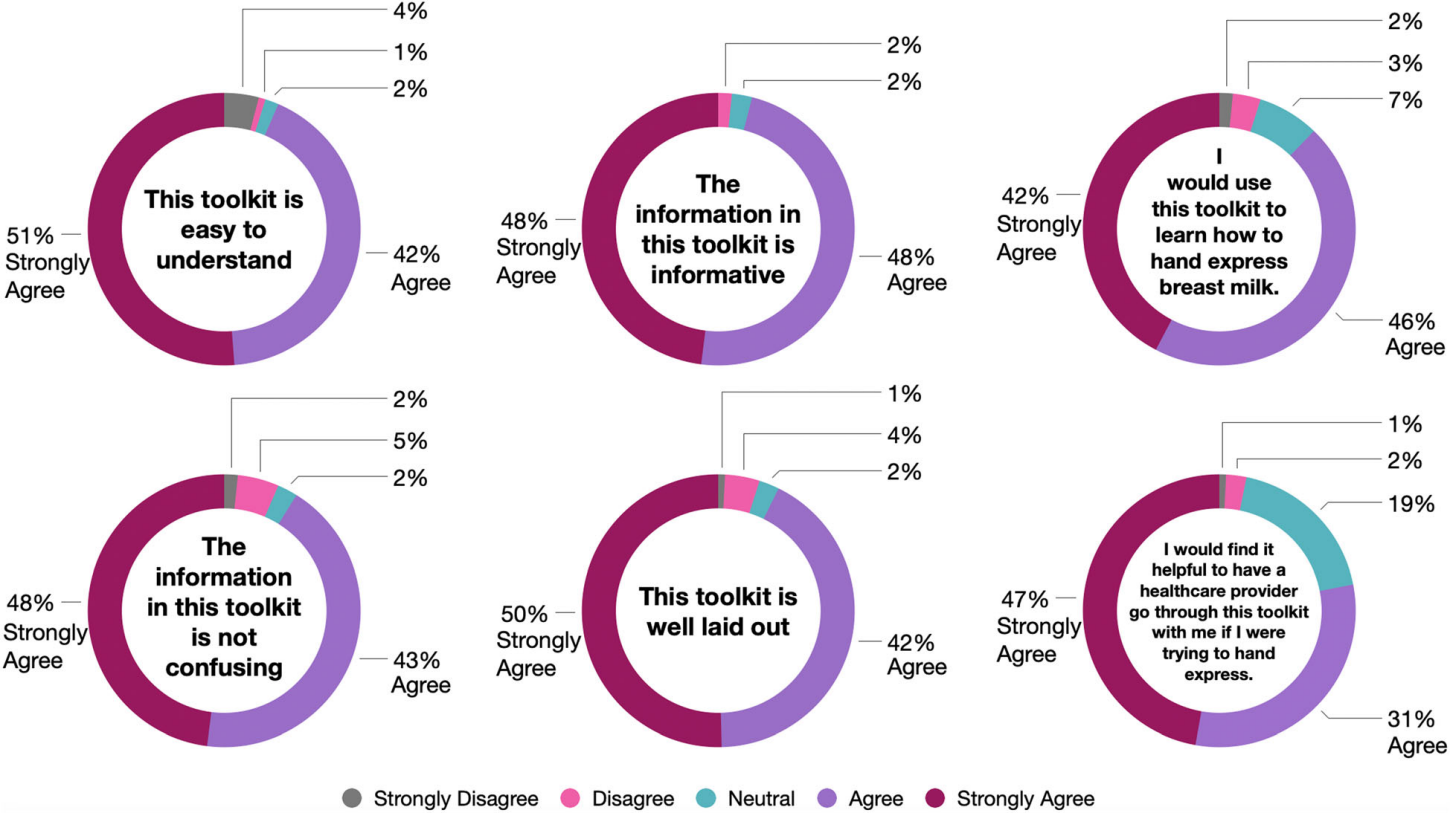
Participant’s overall feedback on the breastmilk hand expression toolkit

Of the 123 participants, a total of 117 (95.1%) had heard of hand expression prior to reviewing the toolkit and 99 (80.5%) had hand expressed before. The feedback regarding the BMHE toolkit, summarized in Fig. 3, was generally favourable; 118 (95.9%) participants said the toolkit was informative, 118 (95.9%) found it utilized inclusive language, 115 (93.5%) said it was easy to understand, 114 (92.7%) said it was well laid-out, 112 (91.1%) said it was not confusing and 105 (85.4%) felt it was visually pleasing. Additionally, 108 (87.8%) participants would use the toolkit to learn how to hand express as well as recommend it to a friend interested in learning how to hand express. Interestingly, 96 (78.1%) participants indicated it would be beneficial to have a healthcare provider go through the toolkit with them if they were trying to learn how to perform BMHE. Of the 76 (61.8%) participants who watched the video included in the toolkit, 73 (96.1%) liked the video as a whole and 69 (90.8%) found it enhanced the information included in the toolkit. When asked about the length of the video, 42 (55.3%) participants who watched it found it to be too long, while 34 (44.7%) participants felt it was not too long, nor too short.

**Fig. 4:**
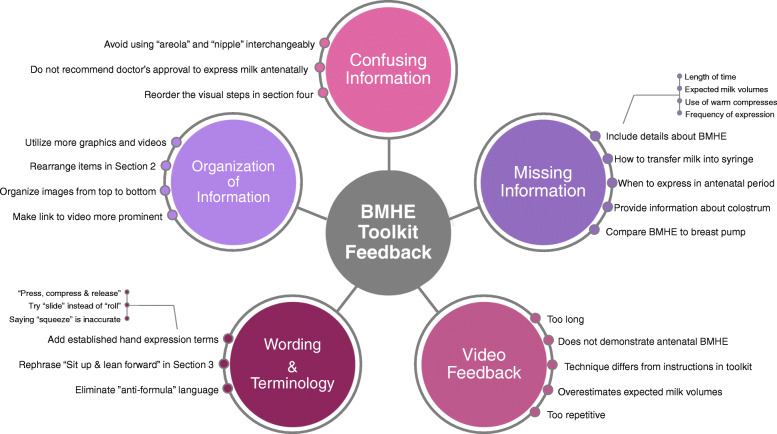
Summary of participant’s written feedback

Main messages from written comments are summarized in Fig. 4. Comments were categorized into one of the following themes: 1) wording and terminology, 2) missing information, 3) confusing information, or 4) organization of information. Excerpts from the comments have been included to illustrate the feedback and suggestions participants included in their submission.

Theme: Wording and terminology

*“I don't agree with [the phrase in section 2] ‘To collect milk before your baby is born to help avoid using formula in the hospital.’ I agree that it's a good point, but I think it would be more positive to phrase it as ‘To collect milk before your baby is born to help with feeding before your milk comes in’ [ . . . ] It shouldn't be already pitting breast vs formula.” – Participant #106, a multiparous individual with breastfeeding and BMHE experience.*

Theme: Missing information

*“Length of time it may take to hand express milk, especially if it doesn't work right away. Also missing information on the expected volumes one can expect [when hand expressing]”. – Participant #200, a primiparous individual who intends to breastfeed and hand express.*

Theme: Confusing information

*"[The phrase] ‘Sit up straight and try leaning forward’ in [section 3] doesn't make sense. You can't be sitting up straight and leaning forward. Perhaps ‘Start sitting down and try leaning forward’ or ‘sitting comfortably, try leaning forward’?” – Participant #137, a multiparous individual with breastfeeding and BMHE experience.*

Theme: organization of information

*“The last two points in the section titled ‘Can I store the milk I collect’ should be combined or reordered. You have to label and store milk before storing. If someone is to follow this like a step-by-step guide, it should be presented in the correct order.” – Participant #100, a primiparous individual who intends to breastfeed and hand express.*

### Participants’ information seeking behaviours

When seeking information about breastfeeding, participants indicated a preference for online resources (58.5%), followed by video resources (22.0%). Participants also identified their preferred sources to find breastfeeding resources: healthcare providers (69.9%), lactational consultants (63.4%), and general internet searches (62.6%). Accordingly, participants felt the BMHE toolkit should be made available in healthcare practitioners’ offices (98.4%), on social media (78.9%), and on websites (78.0%) to ensure widespread distribution. A minority of participants (13.0%) also suggested including the BMHE toolkit in packages provided to parents in the birthing unit, attached to breast pump manuals, and in the aisles of department stores where common baby supplies are purchased.

## Discussion

In this study, a novel educational toolkit that teaches BMHE was developed in consultation with healthcare providers, lactation consultants, and individuals with recent breastfeeding experience, and evaluated by the obstetrical population. Our results suggest that this toolkit was easy to understand, well laid-out, clear, and visually appealing. Participants indicated a preference for online resources and for breastfeeding resources that were provided by healthcare providers.

A key finding in this study was that the majority of participants had heard of hand expression before (95.1%) and had hand expressed their own breastmilk (80.5%). This is a high prevalence of BMHE when compared to other studies that have reported approximately 60% of surveyed participants had experience with hand expression [32]. Our findings may be explained by the fact that the survey was distributed in Facebook groups and shared on Twitter accounts that are frequented by healthcare practitioners who have likely been exposed to BMHE during their training or through personal experience. Furthermore, the number of participants who had exclusively breastfed their infant until six months of age (45.8%) and who had discontinued breastfeeding within the infant’s first month of life (14.6%) were higher and lower, respectively, when compared to what has been previously reported [13, 14]. The higher rates of exclusive breastfeeding and the lower rates of early discontinuation may be explained the fact that our sample was highly educated and overwhelmingly English-speaking. It is likely that these participants were able to seek out timely breastfeeding [33] support and utilize the many breastfeeding resources that are readily available in English to overcome their challenges.

Our novel BMHE toolkit will be revised based on participant feedback and the anticipated revisions are outlined here. In Section 1 (What is hand expression?), participants indicated that they were confused as to why they needed approval from a healthcare provider before performing hand expression in the antenatal period. As such, the second part of the second bullet point in Section 1 that encourages BMHE in the antenatal period will be removed from the toolkit. This is an important revision to highlight as the goal of the toolkit is not to provide education about when to perform BHME, but instead to provide education to individuals on how to effectively perform BMHE. When the toolkit is recommended, whether in the antenatal or postnatal period, will be determined on a case-by-case basis between individuals and their healthcare providers. Next, the language in Section 2 (Why hand express?) that certain participants felt was discouraging the use of human milk supplements, will be completely eliminated. Third, the order of the step-by-step instructions in Section 4 (How do I hand express?) will be reorganized to allow readers to read the images from top to bottom, (i.e., step two will be placed directly beneath step one, instead of to the right of step one). The instructions to describe BMHE in Section 4 will also be rephrased to match the technique outlined in the Global Health Media video [28, 29]. Finally, changes will be made to all sections of the toolkit to ensure that consistent terminology is employed (i.e., any mention of ‘nipple’ will be changed to ‘areola’), instructions are clear and concise (i.e., ‘sit up and learn forward’ will be changed to ‘lean forward’), and established hand expression terminology that is in keeping with the video is utilized (i.e., ‘squeeze’ will be changed to ‘compress’).

Ultimately, the data collected in this survey will be used to create a revised toolkit and to develop and implement tailored strategies to optimize its use. An important consideration prior to widespread distribution of the toolkit is that 78.1% of participants indicated they would find it helpful to have a healthcare practitioner go through the toolkit when learning how to hand express. This finding is important as it suggests that the toolkit on its own may not replace the time requirement that healthcare providers need to spend teaching BMHE in clinical settings but will facilitate this discussion. Accordingly, it may be important to provide healthcare providers with BMHE training, through an online module for example, to ensure that they are correctly teaching their patients this skill. Implementing Baby-Friendly Initiatives (BFI) at local and national hospitals, birthing centres, and community health centres that aim to “protect, promote and support breastfeeding in Canada” will also encourage uptake of the BMHE toolkit [12, 34].

This study is not without limitations. First, the survey response rate of 41% was somewhat lower than the reported response rate for similar electronic surveys of healthcare providers [35]. This may be attributed to the surge in digital technologies that has manifested as a result of the COVID-19 pandemic [36]. Second, the respondents of the survey were not representative of the population in that the majority of participants were Caucasian, English-speaking and highly educated. Therefore, our results are likely biased as we are missing important feedback from Black, Indigenous and people of colour, non-English speakers, individuals with lower education or reading levels and potentially individuals from vulnerable populations. To overcome this, additional focused feedback will be sought from consenting individuals when the toolkit is distributed in local clinical care settings or utilized in future trials where BMHE is being taught. Despite this limitation, when developing patient-facing materials, it is important to seek feedback from the target audience and modify the information accordingly to ensure it is up-to-date, accessible, and informative [37]. Finally, the survey did not evaluate the toolkit’s ability to correctly teach participants how to hand express breastmilk. As such, to improve the quality of this educational tool, it would be important to collect additional feedback and determine whether the steps included in the BMHE toolkit are effective at teaching individuals how to hand express correctly.

## Conclusions

Overall, our BMHE toolkit was well-received and feedback from respondents will be used to address inconsistencies and concerns raised by participants. Our findings provide insight into current breastfeeding practices as well as breastfeeding information-seeking patterns of respondents, which include preferred modality-type and location of information. Although the toolkit was developed in partnership with an expert working group, composed of internationally board-certified lactation consultant, a maternal-fetal-medicine specialist, a general internist, and an endocrinologist, as well as individuals with recent breastfeeding experience, a necessary next step will include evaluation by external experts not involved in the study. Breastfeeding resources should be developed in partnership with both lactational experts and patient partners and evaluated among a diverse group of patient users to ensure that the content is informative, the language is accessible, and that the resource as a whole meets the needs of the target population.

## Supplementary Information

**Additional file 1.** Two-part survey.

## Data Availability

The datasets generated and analysed during the current study are available from the corresponding author upon request.

## Abbreviations

BMHE: Breastmilk Hand Expression.
