# Supplementary material for: Evaluation of a breastmilk hand expression toolkit: the M.I.L.K survey study
Source: Int Breastfeed J. 2022 Jan 15;17:8. doi: 10.1186/s13006-021-00448-3 (PMC8760591; doi:10.1186/s13006-021-00448-3)
Supplement: Supplementary file 1 — Additional file 1. Two-part survey. [file 13006_2021_448_MOESM1_ESM.pdf]

Screening Questions

1. Are you 18 years old or more?
   - ❑ Yes
   - ❑ No

If no, survey ends.

2. With which of the following do you most identify?
   - ❑ I am currently pregnant and intend to feed my child breast milk
   - ❑ I am currently feeding my child breast milk
   - ❑ I fed my child breast milk within the last year
   - ❑ I do not identify with any of these options

If "I do not identify with any of these options", survey ends.

Part 1

1. Where do you currently live?
   - ❑ In the Ottawa/Gatineau region
   - ❑ Outside of Ottawa/Gatineau but in Ontario
   - ❑ Outside of Ontario, but in Canada
   - ❑ Outside of Canada

2. How did you hear about this survey? (select all that apply)
   - ❑ I saw a poster
   - ❑ Social media (e.g. Facebook or Twitter)
   - ❑ Word of Mouth
   - ❑ My healthcare provider asked me to fill out the survey
   - ❑ Other: ___________________

3. How old are you? __________ years

4. You may belong to one or more ethnic or cultural groups. To which group(s) do you most closely identify with? (select all that apply)
   - ❑ Indigenous (First Nations, Metis, or Inuit)
   - ❑ Caucasian
   - ❑ Chinese
   - ❑ Black
   - ❑ Filipino
   - ❑ Latin American
   - ❑ Japanese
   - ❑ Arab
   - ❑ Southeast Asian (e.g. Vietnamese, Cambodian, Laotian, Thai)
   - ❑ West Asian (e.g., Iranian, Afghan)
   - ❑ Korean
   - ❑ Prefer not to answer
   - ❑ Other: _____________________

5. Which of the following language(s) do you commonly speak or read at home? (select all that apply)
   - ❑ English
   - ❑ French
   - ❑ Chinese (e.g. Cantonese, Mandarin, or other dialects)
   - ❑ Tagalog (Pilipino, Filipino)
   - ❑ Portuguese
   - ❑ Somali
   - ❑ Arabic
   - ❑ Spanish
   - ❑ Italian
   - ❑ Panjabi (Punjabi)
   - ❑ German
   - ❑ Urdu
   - ❑ Other: ________________

6. What is your highest level of education?
   - ❑ Grade school
   - ❑ High school
   - ❑ College diploma/undergraduate degree (attended but not yet completed)
   - ❑ College diploma/undergraduate degree (completed)
   - ❑ Graduate/professional school
   - ❑ Prefer not to answer

7. Are you currently pregnant?
   - ❑ Yes
   - ❑ No

   If yes, when is your due date? ______ (dd-mm-yyyy)

8. How many times have you been pregnant? ______

9. How many children have you delivered? _______

10. What feeding methods are you currently using, or did you use to feed your infant after your most recent pregnancy? (select all that apply)
    - ❑ Directly from my breast
    - ❑ Breast milk collected from a pump
    - ❑ Donor breast milk
    - ❑ Formula or milk supplements
    - ❑ Other:
    - ❑ N/A - No breast milk
    - ❑ N/A - I am currently pregnant with my first child

    If other, please specify: ______

    If "N/A" proceed to #22

11. Did you feed your infant breast milk **ONLY** for any length of time (no formula, milk supplements, or solids)?
    - ❑ Yes, I am currently still feeding my child breast milk **only**
    - ❑ Yes, I am not currently feeding my child breast milk **only** but I did at one point
    - ❑ No, I did not ever feed my child breast milk **only**.

If yes proceed to #12 + #13

12. How long has it been so far?
    __ days
    __ wks
    __ months

13. How much longer are you planning to feed your infant breast milk only?
    Until my infant is
    __ days
    __ wks
    __ months

If yes proceed to #14 + #15

14. How long did you feed your infant breast milk only?
    __ days
    __ wks
    __ months

15. How long did you plan on feeding your infant breast milk only?
    __ days
    __ wks
    __ months

16. If you used a pump to collect your breast milk, on average, how often did you use the pump?
    - ❑ I pumped once a day
    - ❑ I pumped 2-4 times a day
    - ❑ I pumped 6-8 times a day
    - ❑ I pumped every feed
    - ❑ I did not use a pump

17. If you used a pump to collect breast milk, for what reason did you use the pump? (select all that apply)
    - ❑ To collect my breast milk so someone else could feed my baby while I was away
    - ❑ Low milk supply
    - ❑ Infant tongue tied/lip tied
    - ❑ For medical reasons. If so, please specify:
    - ❑ For convenience (e.g. easier, personal preference)
    - ❑ Difficulty latching
    - ❑ Breastfeeding pain
    - ❑ Nipple anatomy (e.g. large or inverted nipples)
    - ❑ I did not use a pump
    - ❑ Other: _________________

    If for medical reasons, please specify: ______

18. Did you receive help for any of the breastfeeding challenges you experienced?

❑ Yes ❑ No

19. What challenges did you face while breastfeeding, if any? (select all that apply)
    - ❑ Low milk supply
    - ❑ Exhaustion
    - ❑ Lack of help or support
    - ❑ Lack of confidence
    - ❑ Infant had poor weight gain
    - ❑ Infant tongue tied/lip tied
    - ❑ Difficulty latching
    - ❑ Pain or discomfort
    - ❑ Engorged/Hard breasts
    - ❑ Nipple anatomy (e.g. very large or inverted nipples)
    - ❑ I did not experience any challenges while breastfeeding
    - ❑ Other

    If other, please specify: ______

20. If <u>yes</u>, where did you receive help from? (select all that apply)
    - ❑ Healthcare provider (e.g., family doctor, obstetrician, nurse, midwife)
    - ❑ Lactation consultant
    - ❑ Family/friends
    - ❑ Support Groups (in-person, online)
    - ❑ Educational handouts/pamphlets
    - ❑ Videos
    - ❑ Books
    - ❑ Internet searches/websites
    - ❑ Other: _________________

21. Did you stop feeding your infant breast milk earlier than you would have liked because of the challenges you experienced?
    ❑ Yes ❑ No ❑ I did not experience any challenges while breastfeeding

22. What types of breastfeeding resources have you consulted before? (select all that apply)
    - ❑ Healthcare provider (e.g., family doctor, obstetrician, nurse, midwife)
    - ❑ Lactation Consultant
    - ❑ Friends and family
    - ❑ Support groups (in-person, online)
    - ❑ General internet searches/websites
    - ❑ Educational handouts/pamphlets
    - ❑ Books
    - ❑ Videos
    - ❑ I have not looked up information on breastfeeding
    - ❑ Other: __________________

23. When seeking information related to pregnancy, breastfeeding and related topics, in what format do you prefer to review this information? Rank the following options from favorite (1) to least favorite (3)
    ❑ Printed resources ❑ Online resources ❑ Videos

24. Have you heard of breast milk hand expression before?
    ❑ Yes ❑ No
    ❑

25. **If <u>yes</u>,** from where did you hear or learn about breast milk hand expression? (select all that apply)
    - ❑ Healthcare provider (e.g., family doctor, obstetrician, nurse, midwife)
    - ❑ Friends and family
    - ❑ Lactational Consultant
    - ❑ Support groups (in-person, online)
    - ❑ Health organization/maternity care websites
    - ❑ General internet searches
    - ❑ Media (news, magazines, social media)
    - ❑ Other: __________________

26. Have you come across any helpful hand expression resources that you would like to share with us?

____

27. Have you ever hand expressed breast milk before?
    ❑ Yes ❑ No

Part 2

Please refer to the one-page toolkit attached here. This toolkit teaches hand expression of breast milk. Please read it carefully and then answer the following questions. You may refer to the toolkit at any time.

Section A: The following questions refer to the toolkit as a whole.

1. The information in this toolkit is easy to understand.

Strongly disagree	Disagree	Neutral	Agree	Strongly Agree

2. This information in this toolkit is not confusing.

Strongly disagree	Disagree	Neutral	Agree	Strongly Agree

3. The information in this toolkit is informative.

Strongly disagree	Disagree	Neutral	Agree	Strongly Agree

4. The information in this toolkit is not offensive.

Strongly disagree	Disagree	Neutral	Agree	Strongly Agree

5. This information in this toolkit is well laid-out.

Strongly disagree	Disagree	Neutral	Agree	Strongly Agree

6. This toolkit is overall visually attractive.

Strongly disagree	Disagree	Neutral	Agree	Strongly Agree

7. This toolkit does not have too many words.

Strongly disagree	Disagree	Neutral	Agree	Strongly Agree

8. I would use this toolkit to learn how to hand express breast milk.

Strongly disagree	Disagree	Neutral	Agree	Strongly Agree

9. I would recommend this toolkit to a friend who wants to learn how to hand express.

Strongly disagree	Disagree	Neutral	Agree	Strongly Agree

10. It would be helpful to have a healthcare provider (e.g. lactational consultant, nurse) go through this toolkit with me in person if I was trying to learn how to hand express breast milk.

Strongly disagree	Disagree	Neutral	Agree	Strongly Agree

11. After reviewing the toolkit, would you consider hand expressing breast milk? (select all that apply)
    - ❑ Yes
    - ❑ No
    - ❑ I did hand express during or after my most recent pregnancy
    - ❑ I do not know

12. Where do you think this type of toolkit should be made available? (select all that apply)
    - ❑ In doctors'/nurses'/midwifery offices
    - ❑ On a website
    - ❑ On social media
    - ❑ Other: ____________________

13. If you have any other feedback about this toolkit, please let us know:

    ____

Section B: The following questions refer to the section titled **"What is Hand Expression"**

14. Is there any information in this section that you did not find useful?
    - ❑ Yes ❑ No

    If yes, please elaborate:______________________________

15. Is there any other information that you would have liked to see in this section?
    - ❑ Yes ❑ No

    If yes, please elaborate:______________________________

16. Is any of the information in this section confusing?
    - ❑ Yes ❑ No

    If yes, please elaborate:______________________________

17. Are there any specific words or sentences in this section that you did not understand?
    - ❑ Yes ❑ No

    If yes, please specify:______________________________

Section C: The following questions refer to the section titled "**Why Hand Expression?"**

18. Is there any information in this section that you did not find useful?
    - ❑ Yes ❑ No

    If yes, please elaborate:______________________________

19. Is there any other information that you would have liked to see in this section?
    - ❑ Yes ❑ No

    If yes, please elaborate:______________________________

20. Is any of the information in this section confusing?
    - ❑ Yes ❑ No

    If yes, please elaborate:______________________________

21. Are there any specific words or sentences in this section that you did not understand?
    - ❑ Yes ❑ No

    If yes, please specify:______________________________

Section D: The following questions refer to the section titled **"How Do I Get Started?"**

22. Is there any information in this section that you did not find useful?
    - ❑ Yes ❑ No

    If yes, please elaborate:______________________________

23. Is there any other information that you would have liked to see in this section?
    - ❑ Yes ❑ No

    If yes, please elaborate:______________________________

24. Is any of the information in this section confusing?

❑ Yes ❑ No

If yes, please elaborate:______________________________

25. Are there any specific words or sentences in this section that you did not understand?

❑ Yes ❑ No

If yes, please specify:______________________________

Section E: The following questions refer to the section titled **"How Do I Hand Express?"**

26. The four steps outlined in this section were easy to understand.

**Strongly disagree**	**Disagree**	**Neutral**	**Agree**	**Strongly Agree**

27. The four steps are logically organized.

**Strongly disagree**	**Disagree**	**Neutral**	**Agree**	**Strongly Agree**

28. The font and spacing of the text and diagrams make it easy to read.

**Strongly disagree**	**Disagree**	**Neutral**	**Agree**	**Strongly Agree**

29. The pictures included helped me to better understand the written words.

**Strongly disagree**	**Disagree**	**Neutral**	**Agree**	**Strongly Agree**

30. If I wanted to hand express breast milk, I would be able to follow the four steps outlined in this section.

**Strongly disagree**	**Disagree**	**Neutral**	**Agree**	**Strongly Agree**

31. Is there any information in this section that you did not find useful?

❑ Yes ❑ No

If yes, please elaborate:______________________________

32. Is there any other information that you would have liked to see in this section?

❑ Yes ❑ No

If yes, please elaborate:______________________________

33. Is any of the information in this section confusing?

❑ Yes ❑ No

If yes, please elaborate:______________________________

34. Are there any specific words or sentences in this section that you did not understand?

❑ Yes ❑ No

If yes, please specify:______________________________

Section F: The following questions refer to the **Instructional Video**

35. Did you watch the video highlighted in the pamphlet?

❑ Yes ❑ No

If "No", survey ends.

36. How did you find the length of the video?

❑ Too short ❑ Not too short or too long ❑ Too long

37. Did you like the video as a whole?
    ❑ Yes ❑ No

    If <u>no</u>, what did you not like? ______________________________

38. Is there a section of the video that you did not find helpful?
    ❑ Yes ❑ No

    If <u>yes</u>, which one? ______________________________

39. Is there a section of the video that you did not understand?
    ❑ Yes ❑ No

    If <u>yes</u>, which one? ______________________________

40. Did the video enhance the information outlined in the toolkit?
    ❑ Yes ❑ No

END OF SURVEY
